# Fracture liaison service utilising an emergency department information system to identify patients effectively reduce re‐fracture rate is cost‐effective and cost saving in Western Australia

**DOI:** 10.1111/ajag.13107

**Published:** 2022-07-10

**Authors:** Charles A. Inderjeeth, Warren D. Raymond, Elizabeth Geelhoed, Andrew M. Briggs, David Oldham, David Mountain

**Affiliations:** ^1^ Department of Rehabilitation & Aged Care Sir Charles Gairdner and Osborne Park Hospital Group Nedlands Western Australia Australia; ^2^ School of Medicine & Pharmacology The University of Western Australia Crawley Western Australia Australia; ^3^ School of Allied Health The University of Western Australia Crawley Western Australia Australia; ^4^ School of Physiotherapy & Exercise Science Curtin University Perth Western Australia Australia; ^5^ Medical Education Unit Western Australia Country Health Service Perth Western Australia Australia

**Keywords:** analyses, cost benefit, fractures, health care economics and organizations, integrated health care systems, osteoporotic, prevention, secondary

## Abstract

**Objectives:**

To assess the benefits of the Emergency Department Information System (EDIS)‐linked fracture liaison service (FLS).

**Methods:**

Patients identified through EDIS were invited to attend an FLS at the intervention hospital, the Sir Charles Gairdner Hospital (SCGS‐FLS). The intervention group was compared to usual care. Retrospective control (RC) at this hospital determined historical fracture risk (SCGH‐RC). Prospective control (PC) was from a comparator, Fremantle Hospital (FH‐PC). The main outcome measures were cost‐effectiveness from a health system perspective and quality of life by EuroQOL (EQ‐5D). Bottom‐up cost of medical care, against the cost of managing recurrent fracture (weighted basket), was determined from the literature and 2013/14 Australian Refined Diagnosis Related Groups (AR‐DRG) prices. Mean incremental cost‐effectiveness ratios were simulated from 5000 bootstrap iterations. Cost‐effectiveness acceptability curves were generated.

**Results:**

The SCGH‐FLS program reduced absolute re‐fracture rates versus control cohorts (9.2–10.2%), producing an estimated cost saving of AUD$750,168–AUD$810,400 per 1000 patient‐years in the first year. Between‐groups QALYs differed with worse outcomes in both control groups (*p* < 0.001).

The SCGH‐FLS compared with SCGH‐RC and FH‐PC had a mean incremental cost of $8721 (95% CI −$1218, $35,044) and $8974 (95% CI −$26,701, $69,929), respectively, per 1% reduction in 12‐month recurrent fracture risk. The SCGH‐FLS compared with SCGH‐RC and FH‐PC had a mean incremental cost of $292 (95% CI −$3588, $3380) and −$261 (95% CI −$1521, $471) per EQ‐5D QALY gained at 12 months respectively. With societal willingness to pay of $16,000, recurrent fracture is reduced by 1% in >80% of patients.

**Conclusions:**

This simple and easy model of identification and intervention demonstrated efficacy in reducing rates of recurrent fracture and was cost‐effective and potentially cost saving.

**Practice Impact**

Osteoporosis has a significant impact on patients and society as a whole due the burden of care implications. Fracture liaison services that are cost effective have the potential to improve identification and management of these patients and reduce this impact.

**Policy Impact**

Fracture liaison service should be incorporated into all health services to identify and treat patients with osteoporosis risk. This is beneficial to both patients and funding providers through cost savings.

## 1 INTRODUCTION

Osteoporosis prevalence is increasing in the ageing population.^1^, ^2^, ^3^, ^4^ The first fracture is often the hallmark of this ‘silent’ disease.^5^ The risk of recurrent fracture increases 20% in 1 year, and is sustained for 10 years.^6^, ^7^, ^8^ Osteoporosis affects more women than myocardial infarction, stroke and breast cancer.^4^, ^9^

We reported that over 10 years, osteoporotic fractures cost the Western Australian (WA) health system (population 2.0–2.6 million) more than AUD$100 million in 2012 in direct hospital costs.^9^ In 2007, the overall costs associated with osteoporosis in Australia (population 22 million) were estimated at $1.9 billion.^10^ Health‐care services for osteoporosis compete for health‐care resources across hospitals, ambulatory care services and residential aged care facilities.^8^, ^9^ Over 2.2 million Australians have an osteoporosis‐related condition and this is predicted to increase to 3 million by 2021.^4^, ^10^ Hip fracture rates are estimated to increase four‐fold by 2051. Current estimates that 75% of Australians have undiagnosed and untreated osteoporosis significantly increases downstream health and economic impacts.^8^, ^11^

Emergency Departments (EDs) represent an important entry point for tertiary hospital services and a logical site for efficiently identifying patients presenting with minimal trauma fractures. Fracture liaison services (FLSs) allow timely identification and management of individuals who present to hospitals with osteoporotic fractures.^2^, ^4^, ^8^, ^12^ Seibel proposed that the FLS model should be established nationwide.^8^ There is unequivocal evidence from Australia,^2^, ^13^, ^15^ and internationally^13^, ^16^, ^17^ demonstrating the clinical and cost–benefit of establishing FLS as a public health‐care strategy.^9^ The Western Australian Model of Care for Osteoporosis similarly recommends their implementation in WA. While local data confirm the clinical benefits, the economic benefits are yet to be established. The objective of this study, therefore, was to determine the cost‐effectiveness of an FLS established in a tertiary hospital.^2^, ^15^

## 2 METHODS

### 2.1 Design

**FIGURE 1:**
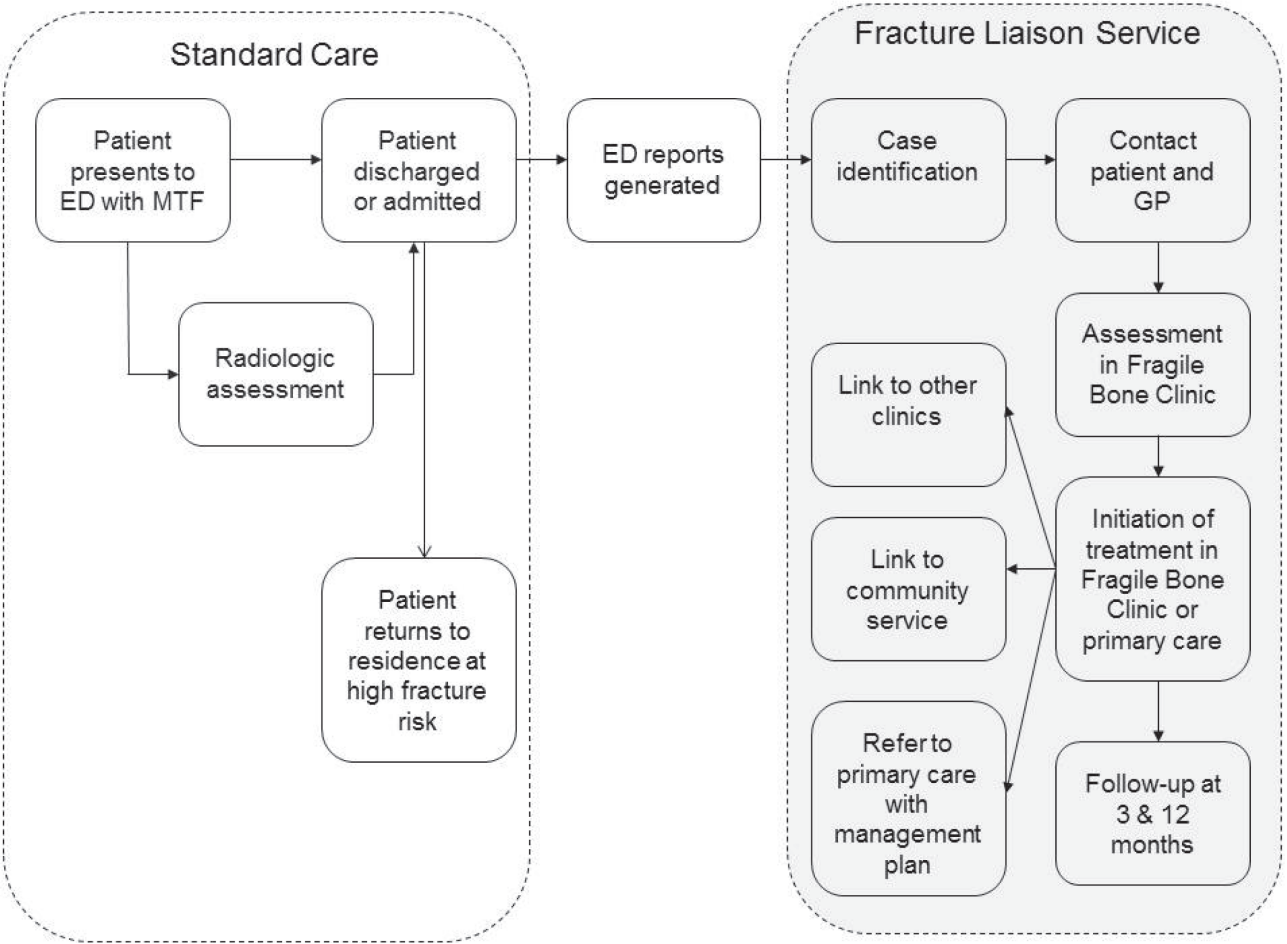
Standard care and the FLS model. FLS, fracture liaison service; ED, Emergency Department; MTF, minimal trauma fracture; GP, general practitioner

A cohort study with two prospective arms: FLS intervention (SCGH‐FLS) and a control comparator hospital site (FH‐PC), without an FLS, was conducted. The design also included a retrospective control arm from the intervention hospital (SCGH‐RC), to determine historical outcomes. The results are reported according to the STROBE statement for cohort studies.^14^ The detailed methods for the source data are as published previously in a companion article (Figure 1).^15^ That study reported health‐related outcomes, while the current study focuses on economic outcomes.

Human research ethics approval was received from the two institutions involved (Sir Charles Gairdner Hospital and Fremantle Hospital); Human Research Ethics Committee – Trial No.: 2012–142. Trial Title: Osteoporotic fracture liaison service – closing the evidence‐practice gap to prevent minimal‐trauma fractures.

### 2.2 Setting

Two tertiary hospital sites where patients presented to the Emergency Department (ED).

### 2.3 Cohort participant selection

Cohorts were identified postdischarge from ED through the Emergency Department Information System (EDIS) within a week of discharge. The prospective SCGH‐FLS intervention cohort recruited participants for 12 months (March 2013 to March 2014), the prospective control (FH‐PC) for 3 months (December 2013 to March 2014) and the retrospective control (SCGH‐RC) for 3 months (October 2012 to January 2013).

Sample size calculation and patient selection were described previously.^15^ It was assumed that the SCGH‐FLS group would have a ≥50% reduction in recurrent fracture rate in the following 12 months based on existing estimates compared to both the prospective and retrospective control cohorts. A sample size estimate determined that each study cohort required 76 participants to achieve a statistical power of 80%, for p < 0.05. Participants aged 50 years or older presenting to ED with minimal trauma fracture were eligible to participate. Patients were identified weekly using ICD‐10‐AM codes from EDIS. A research assistant determined eligibility for review at the FLS. Baseline characteristics such as age, gender and fracture types, are as described previously.^15^

### 2.4 Intervention

The SCGH‐FLS group received FLS outpatient care and the prospective and retrospective control cohorts received usual care.^15^

### 2.5 Outcomes

Basic demographic and clinical information were captured from EDIS, phone or face‐to‐face interview and medical records. Medical care utilisation was assessed at 3 and 12 months utilising a standard schedule. Data collected included investigations, treatments, falls and fractures, medical practitioners or ED visits and quality of life (EQ‐5D).^15^ This was verified through medical reports.^15^

### 2.6 Statistical analysis

Data analysis was conducted with SPSS V. 22. Summary statistics were calculated for baseline, 3 and 12 months. Categorical variables were described with a count and percentage, and proportional differences between groups were assessed with Pearson’s χ^2^ test. Continuous variables, after testing for normality, were presented as a mean and standard deviation. Quality‐of‐life measures (EQ‐5D) were assessed using a GLM repeated measures ANOVA. The incidence of recurrent fracture is presented per 1000 person‐years.

### 2.7 Costs related to patient care postfracture

Costs for this study were determined in a bottom‐up approach wherever practicable (Table S1).

Cost of serological investigations were calculated to reflect ‘best‐practice’ osteoporosis management protocols^18^, ^19^ and priced according to the Medicare Benefits Schedule (MBS – Category 6 – 2013)^21^ in Australian dollars. If a patient indicated that they undertook blood tests related to bone health, it was assumed and costed to include the full bone biochemistry (2013 MBS codes 66512, 66608 and 66695). The weighted cost was estimated at $87.25. This was deliberately calculated as an overestimation to provide the worst‐case scenario cost estimation. Bone densitometry testing and spinal x‐ray were priced at $87.05 and $77.00 respectively (Medicare Benefits Schedule codes).^21^

Treatment of osteoporosis from the payer's perspective only included treatments subsidised by the MBS. Costs borne by patients were not considered in this economic model. The cost of anti‐resorptives was determined from their mean, annualised price in 2013 (AUD$439.83).

The FLS appointment costed $150.00 per patient for a ≤20‐minute appointment with a medical registrar, nursing time and administrative support. A private specialist appointment costed $150.90 (Medicare 2013).^9^, ^21^ The initial general practitioner appointment postfracture costed $70.65 and $36.30 for follow‐up appointments (Medicare Benefits Schedule – Category 1 – December 2013).^21^

The recurrent fracture cost was determined via a top‐down approach based on Cooper et al.^4^ and inflated to 2013 prices in Australia ($8651). Sensitivity analysis to assess a more expensive service utilised the Australia Refined Diagnosis Related Groups (AR‐DRG 2013/14) cost for a pathological fracture ($10,151).

Costs of the FLS co‐ordinator were factored into the cost‐effectiveness analysis, that is, $165,000 for 2 years, that is, $115.00 per patient contact (initial screen: *n* = 714; 3 months: *n* = 241; 12 months: *n* = 202).

### 2.8 Cost‐effectiveness analysis

A cost‐effectiveness analysis from the health system perspective and quality of life (EQ‐5D) was calculated. Recurrent fracture risk was calculated at 3 and 12 months. The recurrent fracture rate and cost for each participant were compared between SCGH‐FLS and control cohorts. We used a ‘bottom‐up’ cost of medical care against the cost of managing recurrent fractures (weighted basket) determined by the literature and 2013/14 AR‐DRG prices to compare the total cost of SCGH‐FLS with control cohorts.

The cohorts were bootstrapped 5000 times to calculate an incremental cost‐effectiveness ratio (ICER) to determine with 95% confidence the incremental cost per 1% reduction in the recurrent fracture rate between two study cohorts. A cost‐effectiveness acceptability curve was generated to guide decision‐makers as to whether or not to adopt the FLS intervention, that is, society's willingness to pay (WTP).

A simple quality‐adjusted life‐year (QALY) gained score was calculated using the changes in EQ‐5D weighted scores (UK version) based on data collected at baseline, 3 and 12 months^15^ and used to measure cost per unit of QALY gained at 12 months.

## 3 RESULTS

The study cohorts were typical of other fragility fracture cohorts described in the literature; mean age 71 years, 70–90% female preponderance and similar fracture profile (20% axial skeleton, 65% upper limb and 15% lower limb). Baseline characteristics were as described previously.^15^

### 3.1 Recurrent fracture rates

Recurrent fracture rates have been described previously (Table 1).^15^ The SCGH‐FLS had a significantly lower proportion of recurrent fractures compared to FH‐PC and SCGH‐RC at 3 months (1.5% vs. 6.7% vs. 8.7%, *p* = 0.03) and 12 months (9.7% vs. 20.0% vs. 20.3%, *p* = 0.02) respectively. Total recurrent fracture rate/1000 person years was calculated for each study cohort. SCGH‐FLS achieved the lowest rate of recurrent fractures at 12 months with 9.7% or 97 recurrent fractures/1000 person‐years compared to 20.0% or 200 recurrent fractures/1000 person‐years (FH‐PC) and 20.3% or 203 recurrent fractures/1000 person‐years (SCGH‐RC) (*p* < 0.001).

**TABLE 1:** Fracture and quality‐of‐life outcomes across baseline, 3 and 12 months

			Group			Test	SCGH‐FLS vs. FH‐PC	FLS‐SCGH vs. SCGH‐RC
			SCGH‐FLS (*n* = 202)	FH‐PC (*n* = 45)	SCGH‐RC (*n* = 92)			
			Mean ± SD or *n* (%)	Mean ± SD or *n* (%)	Mean ± SD or *n* (%)			
Recurrent fracture events	By 3 months follow‐up	Patient fractured since baseline	3 (1.5)	3 (6.7)	8 (8.7)	aOR (95% CI)	0.30 (0.06, 1.54)	0.19 (0.05, 0.76)
		Total fractures in the cohort	3 (1.5)	3 (6.7)	9 (9.8)	RR (95% CI)	0.22 (0.04, 1.30**)**	0.15 (0.03, 0.54)
	By 12 months follow‐up	Patients fractured since baseline	17 (8.1)	8 (17.3)	17 (18.3)	aOR (95% CI)	0.40 ( 0.16, 1.01)	0.38 (0.18, 0.79)
		Total fractures in the cohort	19 (9.5)	10 (22.2)	20 (21.7)	RR (95% CI)	0.42 (0.20, 0.95)	0.43 (0.23, 0.82)
		Fractures per 1000 patient‐years	97	200	203	RR (95% CI)	0.49 (0.38, 0.62)	0.48 (0.37, 0.61)
EQ‐5D United Kingdom Weighted Index Score (0–1)		3 Month	0.60 ± 0.34	0.73 ± 0.12	0.82 ± 0.13	*t*‐test	<0.001	0.003
		12 Month	0.69 ± 0.32	0.65 ± 0.34	0.69 ± 0.30	*t*‐test	0.364	0.097
		Change from 3 to 12 Months	+15%	−11%	−16%	ANOVA	0.139	0.001
EQ‐5D Health State VAS (0–100)		3 Months	57.11 ± 34.67	69.64 ± 16.33	74.45 ± 20.05	t‐test	<0.001	<0.001
		12 Months	73.48 ± 20.05	67.94 ± 19.64	75.21 ± 18.17	t‐test	0.948	0.482
		Change from 3 to 12 Months	+29%	−2%	+1%	ANOVA	0.540	1.00

*Note*: Mean ± SD: Mean and Standard Deviation;

Abbreviations: aOR, adjusted odds ratio derived from a multivariate logistic regression model that is adjusted for age and sex; RR, rate ratio.

ANOVA – multiple comparisons with Bonferroni adjustment of EQ‐5D UK weighted score and EQ‐5D Health State VAS across study groups.

### 3.2 Quality of life and health perception measures

The QOL outcomes are as described previously (Table 1).^15^ The EQ‐5D UK weighted scores improved for the SCGH‐FLS from 3 to 12 months by 15%, but deteriorated in the FH‐PC(−11%) and SCGH‐RC (−16%). Within‐ and between‐group analyses demonstrated a significant difference in the EQ‐5D UK weighted score across groups and time points, *F*(2, 309) = 116.92, *p* < 0.001.

Mean changes in EQ‐5D health state visual analogue scale (VAS) from 3 to 12 months improved by 29% for the SCGH‐FLS group compared to no change in the FH‐PC and SCGH‐RC. Within‐ and between‐group analyses demonstrated a significant difference in the EQ‐5D score across groups, *F*(2, 321) = 390.62, *p* < 0.001.

### 3.3 Health economic analysis

#### 3.3.1 Recurrent fracture rates

**FIGURE 2:**
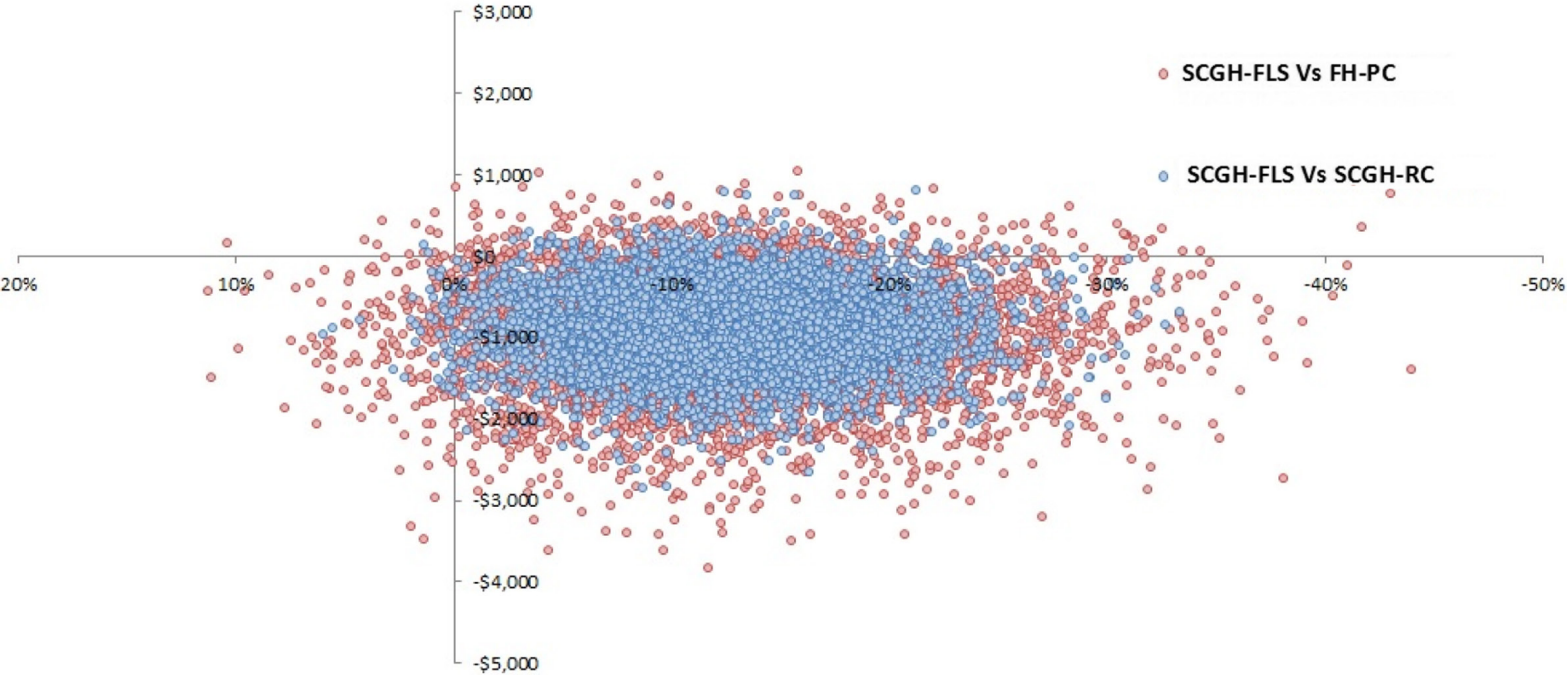
Scatter plot of cost‐effectiveness and fracture rate reduction. SCGH‐FLS individual patient data compared to control cohorts. *x*‐axis: fracture risk reduction; *y*‐axis: cost savings

Individual patient data of cost‐effectiveness demonstrated a reduction in both fracture and costs (Figure 2). We calculated the cost to the payer per 1% reduction in recurrent fracture risk/1000 patient‐years for the SCGH‐FLS intervention compared to the SCGH‐RC and FH‐PC. A 1% reduction in recurrent fracture risk attributable to the establishment of the SCGH‐FLS had an incremental cost of $8721 (95% CI −$1218, $35,044) and $8974 (95% CI −26,701, $69,929) compared to the SCGH‐RC and FH‐PC respectively (Table 2).

**TABLE 2:** Incremental cost analysis of FLS

2a. Incremental cost for a 1% reduction in recurrent fracture rate at 1 year			Incremental cost‐effectiveness		
			Mean	Lower 95%	Upper 95%
Recurrent fracture rate	SCGH‐FLS vs. SCGH‐RC	Payer perspective	$8721	−$1218	$35,044
		Payer light	$6880	−$447	$38,511
		AR‐DRG 2013/14	$10,626	−$621	$46,919
	SCGH‐FLS vs. FH‐PC	Payer perspective	$8974	−$26,701	$69,929
		Payer light	$7700	−$26,477	$69,074
		AR‐DRG 2013/14	$14,161	−$48,551	$79,808

2b. Incremental cost for an increase in 1 EQ‐5D QALY at 1 year			Incremental cost‐effectiveness ratio		
			Mean	Lower 95%	Upper 95%
QALYs gained	SCGH FLS vs. SCGH‐RC	Payer perspective	$292	−$3588	$3830
		Payer light	$272	−$3189	$4016
		AR‐DRG 2013/14	$1254	−$5498	$6577
	SCGH FLS vs. FH‐PC	Payer perspective	−$261	−$1541	$471
		Payer light	−$214	−$1672	$710
		AR‐DRG 2013/14	−$379	−$1915	$863

The table demonstrates the average cost to the payer, with a 95% confidence Interval, to reduce recurrent fracture rates by 1% or gain 1 EQ‐5D QALY at 1 year for the SCGH‐FLS versus the SCGH‐R and FH service. The payer perspective is the unadulterated model based on the rates of investigations, treatments, clinician time and additional costs seen throughout the study. The payer light is a deterministic sensitivity analysis which excluded the cost of spinal x‐rays. The AR‐DRG 2013/14 model is, again, a deterministic sensitivity analysis which uses a weighted cost of fractures produce by government estimates.

Based on reduced absolute re‐fracture rates versus control cohorts of 9.2–10.2% equating to a reduction of 100 fractures per 1000 patient‐years, for an investment of AUD$16,000 offset against costs associated with treating recurrent fracture, we estimated cost saving of $750,168–$810,400/1000 patient‐years in the first year.

#### 3.3.2 Quality‐adjusted life‐years gained

Individual patient data of SCGH‐FLS cost‐effectiveness versus QALY gained was used to estimate the cost to the payer per 1 EQ‐5D QALY gained by 12 months postfracture. An incremental cost per EQ‐5D QALY gained 12 months postfracture was $292 (95% CI −$3588, $3380) compared to the SCGH‐RC −$261 (95% CI −$1521, $471) and the FH‐PC (Table 2).

#### 3.3.3 Cost‐effectiveness acceptability (willingness to pay)

**FIGURE 3:**
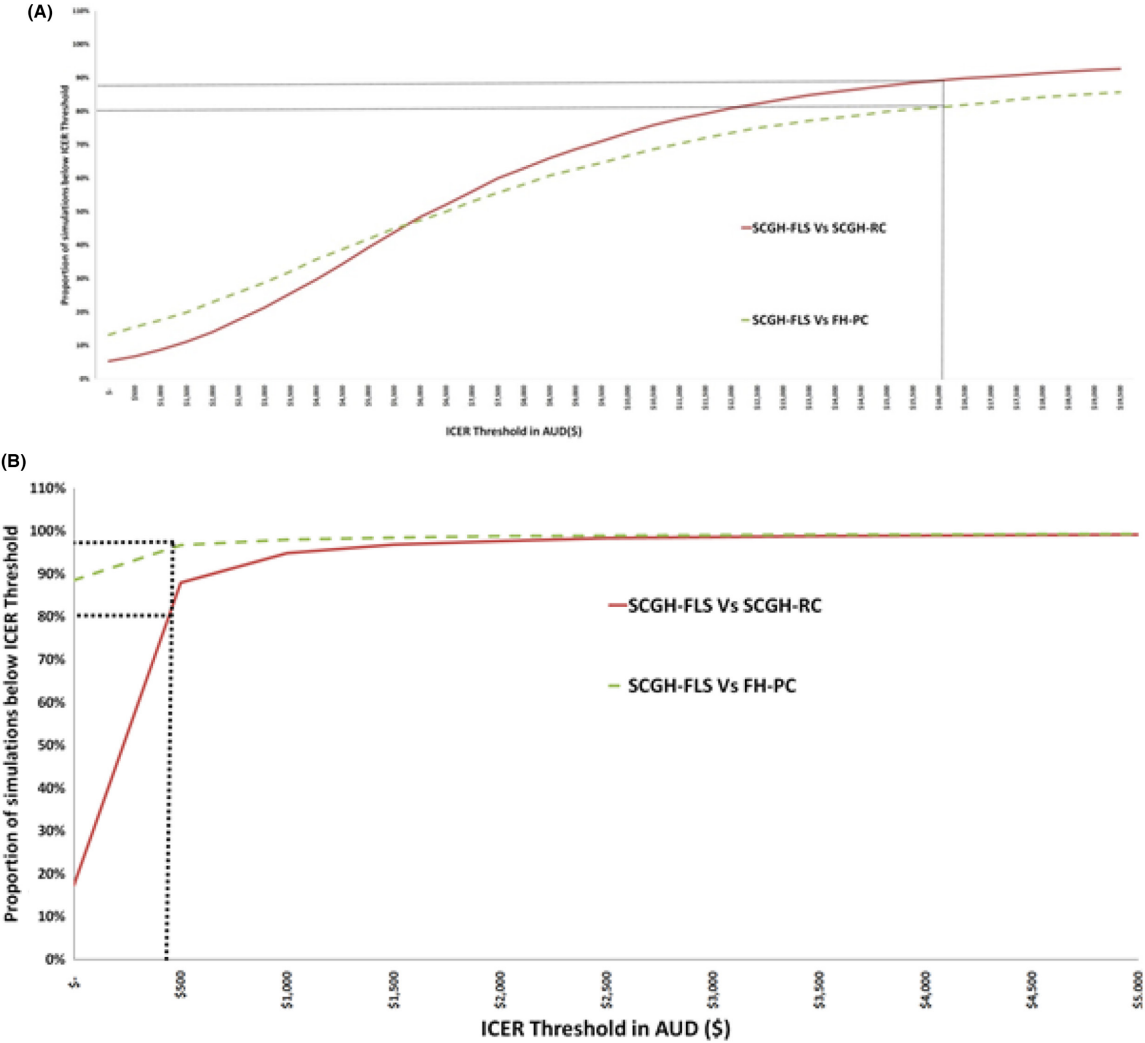
Cost‐effectiveness acceptability curve for (A) fracture reduction and (B) EQ‐5D QALY at 12 months. (A) Cost‐effectiveness acceptability curves for recurrent fracture rates illustrates that at a willingness to pay of approximately $16,000 (*x*‐axis) the payer expects to see a reduction in the recurrent fracture rate of 1% in 90% (*y*‐axis) of simulations for the SCGH‐FLS vs. SCGH‐RC model and in 80% (*y*‐axis) of simulations for the SCGH‐FLS vs. FH‐PC model. (B) Cost‐effectiveness acceptability curves for QALYs gained by 12 months illustrates that at a willingness to pay of approximately $500 (*x*‐axis) the payer expects to see a QALY gained in 80% (*y*‐axis) of simulations for the SCGH‐FLS vs. SCGH‐RC model and in 98% (*y*‐axis) of simulations for the SCGH‐FLS vs. FH‐PC model

The cost‐effectiveness acceptability curve demonstrates the financial threshold at which simulations will produce either a 1% reduction of the recurrent fracture rate or one additional EQ‐5D QALY gained by 12 months. For a willingness to pay of approximately $16,000, the payer can expect that the FLS intervention would deliver a 1% reduction of the recurrent fracture rate in 80–90% of simulations. This equates to 10/1000 fewer ED presentations for fragility fracture per annum, which translates to savings of approximately $8651–$10,151 per fracture, making the SCGH‐FLS intervention cost‐effective (Figure 3A).

For a willingness to pay of $500, the payer can expect an improvement in 1 EQ‐5D QALY by 12 months in 95–99% of simulations. This compares favourably with QALYs gained in other conditions including breast cancer^20^ (Figure 3B).

## 4 DISCUSSION

We evaluated the economic benefits of implementation of an FLS model in a major tertiary hospital compared to usual care. This unique model utilised an existing ED database to identify patients, reducing the inefficiencies associated with traditional models that have the ED referred patients (low referral rates) or have an FLS liaising directly with the ED (labour intensive and expensive).^2^ This method ensured more complete patient capture based on 24/7 presentations as opposed to case finding. Furthermore, the implementation of a multidisciplinary model of care, combining fall prevention with osteoporosis care, resulted in earlier than expected benefits within 12 months.

We demonstrated that bridging the gap between patients and clinicians for postfracture management and utilising an FLS is both cost‐effective and a sustainable solution for the payer. It also reduces the downstream burden on the health system as a consequence of inadequate management of patients with minimal trauma fracture. This FLS cohort delivered an absolute reduction of recurrent fracture rates by approximately 10% or a relative reduction of more than 50% by 12 months. This represents a prevention of 100 recurrent fractures/1000 patient‐years, representing a cost saving in direct health‐care costs, based on our conservative, weighted cost per fracture, of approximately AUD$750,000/1000 patients treated per annum.

These results are consistent with the findings from other similar FLS cost‐effectiveness studies, supporting the proposition that more intensive models are significantly more effective in the prevention of recurrent fractures.^22^, ^23^ Our FLS was costed in a natural, bottom‐up approach using local costs where possible, and the weighted cost of a fracture was determined from the literature or government AR‐DRGs. Both costs would be deemed conservative compared to other published costs.^4^

International studies have reported the benefit of an FLS with an incremental cost per QALY‐gained of −$1083.^24^ A similar study to ours in Canada demonstrated benefit in 6/100 patients (4 hip)^25^ and modest improvement in QALYs and cost saving of CAD260,000 (2009) within 12 months.^25^ A Glasgow study concluded that FLS prevented 18 fractures (11 hip) for a net saving (GBP21,000).^26^ The Ontario Fracture Clinic Screening Program for an investment of CAD83,000/1000 patients screened was cost‐effective and improved QALY by 4.3 years at a cost of CAD19 per QALY gained.^27^ An Australian simulated cost‐effectiveness analysis over 10‐year predicted that the FLS could reduce re‐fracture rates by 80%, improve QALYs by 0.089 (95% CI 0.059–0.122) with an ICER of $17,291 per QALY‐gained.^4^

The costs reported in our study are both conservative and in line with those proposed in the wider literature by other FLS programs. Recurrent fracture rate and associated costs of re‐fracture are the major determinants of cost to the *payer* in all models, which overwhelms the cost of assessment or treatment. From a societal perspective, it is reported that the indirect costs of fragility fractures are reported to be triple those of the direct costs. Hence, all models understate the true cost of fracture events.^4^

A strength of this study is that it utilised actual costs and, where these were uncertain, overestimated them. It factored in extra human resource costs to better reflect the actual costs of care. This project was a seminal piece on the cost‐effectiveness of FLS in the context of shrinking health‐care budgets. We utilised a real‐life model rather than a simulation (Markov modelling) for more realistic cost‐effectiveness within a short time frame (12 months). Subsequent studies should aim to confirm longer term (beyond 12 months) economic benefits.

There are some potential limitations in our study. Firstly, we used a retrospective cohort from the same hospital and a parallel cohort from a similar hospital as control groups instead of a randomised control study which would be the gold standard. This was not possible in this study due to ethical considerations. Furthermore, for this reason the improvement in the QALY between groups is difficult to attribute to fracture reduction, but rather to the higher intensity of intervention and fall risk management in the intervention group. Nevertheless, this confirms the benefit of such a program. The selective nature of the intervention group (largely self‐selected due to voluntary participation) may also introduce a bias which may limit the external validity to all patients as the frailest patients and the most well may have declined participation. There may be recall bias as patients were interviewed at 3 and 12 months only. To compensate for this potential weakness, we reviewed clinical records. Participant management costs related to their osteoporosis were calculated in a ‘bottom‐up’ approach based on study data collected. A further limitation is that indirect costs borne by patients and carers were not collected, underestimating the true overall cost and potential savings. Missing information or additional unrecorded interventions cannot be accounted for in the cost analysis. The FLS intervention was directed at case finding rather than clinician upskilling which may explain the lower rates of pharmacological treatment. Targeted education of treating clinicians may improve outcomes further.

## 5 CONCLUSIONS

An FLS established in a metropolitan tertiary hospital utilising an existing EDIS to identify patients who were managed in a multidisciplinary model of care was successful in reducing recurrent fracture, improved QOL, was cost‐effective and had acceptable costs. If this program was implemented in metropolitan regions, the health system would stand to save approximately AUD$1 million/1000 person‐years by 12 months through delivering a 50% relative reduction (10% absolute risk reduction) in recurrent fracture rates compared to the current care.

## CONFLICTS OF INTEREST

No conflicts of interest declared.

## Supporting information


Table S1

## Data Availability

Data available on request from the authors.
