# Supplementary material for: Fracture liaison service utilising an emergency department information system to identify patients effectively reduce re‐fracture rate is cost‐effective and cost saving in Western Australia
Source: Australas J Ageing. 2022 Jul 10;41(3):e266–75. doi: 10.1111/ajag.13107 (PMC9545318; doi:10.1111/ajag.13107)
Supplement: Supplementary file 1 — Table S1 [file AJAG-41-e266-s001.docx]

**Table S1: Itemised costs utilised in the current project**

| **Cost Table - Payer Perspective** | **2013/14 Prices (AUD$)** |
| --- | --- |
| **Investigations** | |
| Blood Work ^a^ | $ 87.25 |
| Bone Mineral Densitometry ^b^ | $ 87.05 |
| Spinal X-ray ^c^ | $ 77.00 |
|  |  |
| **Treatments** | |
| Calcium per annum ^d^ | $ 98.29 |
| Vitamin D per annum ^e^ | $ 70.70 |
| Antiresorptives per annum (mean) ^d^ | $ 439.83 |
|  |  |
| **Clinician Time** | |
| Fragile Bone Clinic appointment ^f^ | $ 150.00 |
| Private Specialist Appointment (Medicare rebate) ^g^ | $ 150.90 |
| GP Visit (post-fracture) ^g^ | $ 70.65 |
| GP Visit (follow-up) ^g^ | $ 36.30 |
| **Additional Costs** | |
| Mean cost of Fracture ^h^ | $ 10,150.88 |
| Research Cost (2013 prices) ^i^ | $ 115.00 |
|  | |
| a MBS Schedule (66512, 66608, 66695) | |
| b Quote from local Gairdner Bone Densitometry (MBS Schedule) | |
| c Medicare Benefits Schedule - Category 5 - from 01 December 2013 (58106) | |
| d PBS - Dispensed Maximum Price per Quantity adjusted from 2015 price | |
| e Online - adjusted from 2015 prices | |
| f Departmental price to host service (2013/14) | |
| g Medicare Benefits Schedule - Category 1 - from 01 December 2013, (110, 732, 2501) | |
| ^h^ Cooper et al. (2011) | |
| i Cost of FLS over 714 patients contacted over 12 months via SCGH Emergency Dept. | |
